# Screening out Biomarkers of *Tetrastigma hemsleyanum* for Anti-Cancer and Anti-Inflammatory Based on Spectrum-Effect Relationship Coupled with UPLC-Q-TOF-MS

**DOI:** 10.3390/molecules28073021

**Published:** 2023-03-28

**Authors:** Jie Xia, Xiuyue Li, Min Lin, Jiani Yu, Zhongda Zeng, Fei Ye, Guanjun Hu, Qiang Miu, Qiuling He, Xiaodan Zhang, Zongsuo Liang

**Affiliations:** 1 Key Laboratory of Plant Secondary Metabolism and Regulation of Zhejiang Province, College of Life Sciences and Medicine, Zhejiang Sci-Tech University, Hangzhou 310018, China; 2 College of Environmental and Chemical Engineering, Dalian University, Dalian 116000, China; 3 Hangzhou Shizhuwu Agricultural Technology Co., Ltd., Hangzhou 311400, China; 4 Hangzhou Fuyang District Agricultural Technology Extension Center, Ltd., Hangzhou 311400, China

**Keywords:** *Tetrastigma hemsleyanum*, UPLC-Q-TOF-MS, anti-cancer, anti-inflammatory, spectrum-effect relationship, biomarkers

## Abstract

*Tetrastigma hemsleyanum* Diels et Gilg. (*T. hemsleyanum*) is an economically and medicinally valuable species within the genus Tetrastigma. However, the material basis of its pharmacological action and the biomarkers associated with its anti-cancer and anti-inflammatory effects are still unclear. Additionally, the *T. hemsleyanum* industry cannot grow because there is a lack of a scientific, universal, and measurable quality control system. This study aimed to explore the chemical basis quality markers related to the anti-cancer and anti-inflammatory effects of *T. hemsleyanum* to establish an effective quality evaluation method. UPLC-Q-TOF-MS^E^ fingerprint profiles of *T. hemsleyanum* from different origins were established. Pharmacodynamic studies used HepG2 and HuH-7 cells and LPS-induced RAW264.7 to evaluate the anti-tumor and anti-inflammatory effects of the active ingredients. The spectrum-effect relationships between UPLC fingerprints and anti-cancer and anti-inflammatory activities were evaluated using PCA and PLSR statistical methods. Moreover, docking analysis was performed to identify specific active biomarkers with molecular targets associated with cancer and inflammation. Chlorogenic acid, quinic acid, catechin, kaempferol 3-rutinoside, apigenin-8-C-glucoside, and linolenic acid were associated with anticancer activity, while chlorogenic acid, quercetin, quinic acid, kaempferol 3-rutinoside, rutinum, apigenin-8-C-glucoside, and linolenic acid were associated with anti-inflammatory activity. The spectrum-effect relationship of *T. hemsleyanum* was successfully established, and the biomarkers for anti-cancer and anti-inflammatory effects were preliminary confirmed. These findings provide a theoretical basis for the elucidation of the substance basis of *T. hemsleyanum* and lay the foundation for its rapid identification, quality control, industrial research, and utilization.

## 1. Introduction

*Tetrastigma hemsleyanum* Diels et Gilg. (*T. hemsleyanum*), also known as Sanyeqing, Jinxiandiaohulu, Shefuzi, or Shilaoshu in China, is a widely used herbaceous plant in traditional Chinese medicine [1,2]. This rare plant grows in subtropical areas of mainland China. It was used to treat fever for the first time in the 3rd century in the book Ming Yi Bie Lu [2]. In the 16th century, the Compendium of Materia Medica focused on the plant’s ability to dispel heat and toxins as well as promote blood circulation [3]. Today, *T. hemsleyanum* is commonly used in folk medicine to treat high fever, infantile febrile convulsion, pneumonia, snake bite, and jaundice. It has been cited in both the Flora of China and the Chinese Pharmacopeia (2015 edition) [3,4,5]. *T. hemsleyanum* root tubers have been used as herbal medicine in Chinese local pharmacopoeia, while its leaves are popularly consumed as a functional tea or dietary supplement in local regions of China [6]. Due to its wide range of pharmacological effects, the plant is cultivated on a large scale.

Currently, systematic chemical profile studies have been conducted for different organs of *T. hemsleyanum*, including roots, stems, and leaves [1]. The chemical constituents of *T. hemsleyanum* are mainly flavonoids and polyphenolic acids, such as kaempferol, apigenin-8-C-glucoside, chlorogenic acid, and catechins [2]. Recent pharmacological studies have shown that kaempferol and other components of *T. hemsleyanum* possess a broad range of activities [7,8]. For example, in a variety of human cancer cell lines, kaempferol has exhibited a wide range of anti-cancer activities, such as apoptosis, cell cycle arrest, downregulation of tissue transformation-related markers, and phosphoinositide 3-kinase/protein kinase B signaling pathways [9,10,11]. In addition, chlorogenic acid has a strong anti-inflammatory effect on the metabolism of arachidonic acid by inhibiting the activation of inflammatory factors such as HIF-1a, ICAM-1, VCAM-1, TNF-α, IL-6, and nuclear factor kappa B (NK-KB) p65. This protects against cerebral ischemia/reperfusion injury and liver fibrosis [12,13]. Due to its remarkable anti-cancer and anti-inflammatory effects, *T. hemsleyanum* has been suggested as a promising option for the treatment of various pathological conditions in clinical settings.

Liver cancer is one of the six most common forms of cancer worldwide, with a very high mortality rate [14]. HepG2 cells originate from a human liver embryonic tumor cell line, have a similar phenotype to hepatocytes, and have been widely used as an in vitro model to evaluate the potential of drug-drug interactions [15,16]. HuH-7 is another hepatocellular carcinoma cell line, which is highly correlated with primary human hepatocytes in gene expression profiles of drug-metabolizing enzymes and transporters [17,18]. Therefore, a large number of studies have been conducted on HepG2 and HuH-7 cell lines to evaluate the induction and reversal of human hepatocellular carcinoma resistance treatment by natural products [19,20]. Inflammation is a defensive response of the body to stimuli triggered by tissue damage, such as invading microbes and tissue injury [21]. Due to the massive secretion of effectors downstream of the inflammatory pathway, inflammatory inducers trigger the production of a large number of various inflammatory cytokines, which in turn alter the function of many tissues and organs [22]. Inflammatory cytokines usually play a role in activating macrophages and promoting cell-mediated immune responses against invasive intracellular pathogens, such as tumor-necrosis factor-α (TNF-α), IL-1, IL-6, and many others [23]. Inflammation can contribute to cancer progression, and conversely, cancer can induce inflammatory processes [24]. Screening of key components from natural products that antagonize inflammatory cells and cytokines, inhibit tumor growth, and enhance anti-cancer immunity will help find new strategies to prevent or treat cancer.

Currently, the effective components of *T. hemsleyanum* are not clearly known, which presents a challenge to the quality control of *T. hemsleyanum* entering the market circulation. Thus, we proposed a strategy to establish the spectrum-effect relationship of *T. hemsleyanum* using UPLC-Q-TOF-MS and activity analysis. The correlations between the common peaks and anti-inflammatory and anti-cancer activities were studied by principal components analysis (PCA) and partial least squares regression (PLSR) statistical methods to further reveal the active substance basis of *T. hemsleyanum.* This provides a basis for exploring the quality markers of the medicinal plant *T. hemsleyanum*, establishing its quality evaluation method, and investigating the mechanism of action of its medicinal components.

## 2. Results

### 2.1. Screening for Biomarkers of Anticancer Activity of T. hemsleyanum in HepG2 Hepatocellular Carcinoma Cells

#### 2.1.1. UPLC-Q-TOF-MS^E^ Fingerprint Profiles of *T. hemsleyanum* Extracts from Different Origins

**Figure 1:**
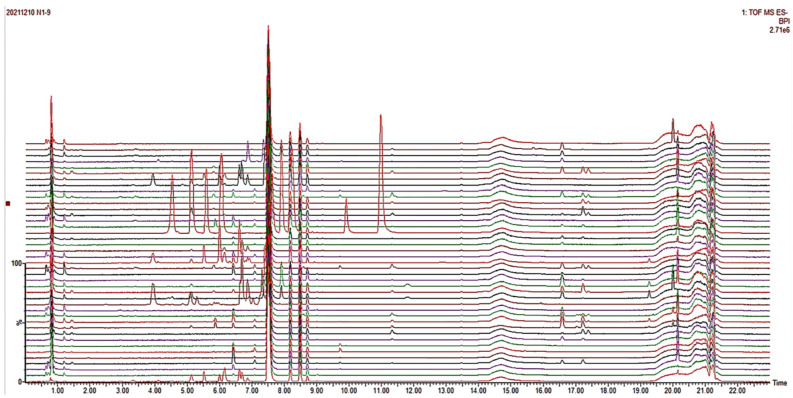
The total ion chromatography (TIC) of *T. hemsleyanum* extracts from different origins.

The total ion chromatography of methanolic extracts of *T. hemsleyanum* from different origins was obtained by UPLC-Q-TOF-MS^E^ (Figure 1). The representative total ion chromatography (TIC) of *T. hemsleyanum* extract is shown in Figure 1. QI was used for automatic peak identification, matching, alignment, extraction, integration, and normalization of the mass spectrometry data. Metabolites were identified using secondary mass spectrometry data, online databases (SciFinder, ChemSpider, and PubMed), and data from previous studies (UNIFI, Waters Corporation). At Rt = 7.50 min, the ion peak m/z 417.2371 [M-H]^−^ of internal standard liquiritin appeared in all samples. This indicates that the instrument has good precision and repeatability and is suitable for fingerprint analysis. The results showed that although there was a certain degree of similarity between the fingerprint spectra of *T. hemsleyanum*, the areas and numbers of component peaks in the total ion flow spectra of different origins were not completely consistent. Apart from some common peaks in the samples, there is significant variation in the chemical composition spectra between different origins. This may be related to some factors, such as growth environment, harvesting season, cultivation method, and processing method. The low similarity between samples from different origins may lead to variability in the activity of the extracts. This provides an experimental basis for subsequent spectrum-effect correlation analysis between the chemical composition spectra and activities of *T. hemsleyanum* extracts.

#### 2.1.2. Anti-Cancer Efficacy Analysis on HepG2 Cells

The CCK-8 method was used to analyze the anticancer effect of samples from different sources. The findings indicated that there was a negative correlation between the concentration of *T. hemsleyanum* extract from different origins and the survival rate of HepG2 cells. The results of Table 1 showed that *T. hemsleyanum* extract had significant anticancer efficacy. A comparative analysis of Table 1 revealed that the anticancer IC_50_ values of most methanol extracts of *T. hemsleyanum* ranged from 100 μg/mL to 600 μg/mL. This indicates that these extracts have good anticancer effects on HepG2 cells, with a concentration trend observed.

Among them, the best anticancer effect on HepG2 was obtained from the methanolic extract of *T. hemsleyanum* Puqian (IC_50_ = 98.7 μg/mL) from Zhejiang Province. *T. hemsleyanum* samples, including FJ-SM (IC_50_ = 127.3 μg/mL), SC-CQ (IC_50_ = 129.4 μg/mL), ZJ-FY (IC_50_ = 150.7 μg/mL), GD-SC (IC_50_ = 171.9 μg/mL), ZJ-QDH (IC_50_ = 178.1 μg/mL), and JX-JGS (IC_50_ = 187.5 μg/mL) showed strong inhibitory effects on HepG2 tumor cells. In contrast, *T. hemsleyanum* (IC_50_ = 983.8 μg/mL), named AH-HS, had a weaker inhibitory effect on HepG2 tumor cells.

The methanolic extract of *T. hemsleyanum* from Puqian, Zhejiang Province, showed the best anticancer effect on HepG2 cells with an IC_50_ value of 98.7 μg/mL. Other *T. hemsleyanum* extracts, such as FJ-SM (IC_50_ = 127.3 μg/mL), SC-CQ (IC_50_ = 129.4 μg/mL), ZJ-FY (IC_50_ = 150.7 μg/mL), GD-SC (IC_50_ = 171.9 μg/mL), ZJ-QDH (IC_50_ = 178.1 μg/mL), and JX-JGS (IC_50_ = 187.5 μg/mL), also exhibited strong inhibitory effects on HepG2 tumor cells. In contrast, *T. hemsleyanum*, named AH-HS, had a weaker inhibitory effect on HepG2 tumor cells, with an IC_50_ value of 983.8 μg/mL. This suggests that the source of *T. hemsleyanum* extract can significantly affect its anticancer efficacy.

#### 2.1.3. Spectrum-Effect Relationship of *T. hemsleyanum* Extracts on HepG2 Anticancer Activity

**Figure 2:**
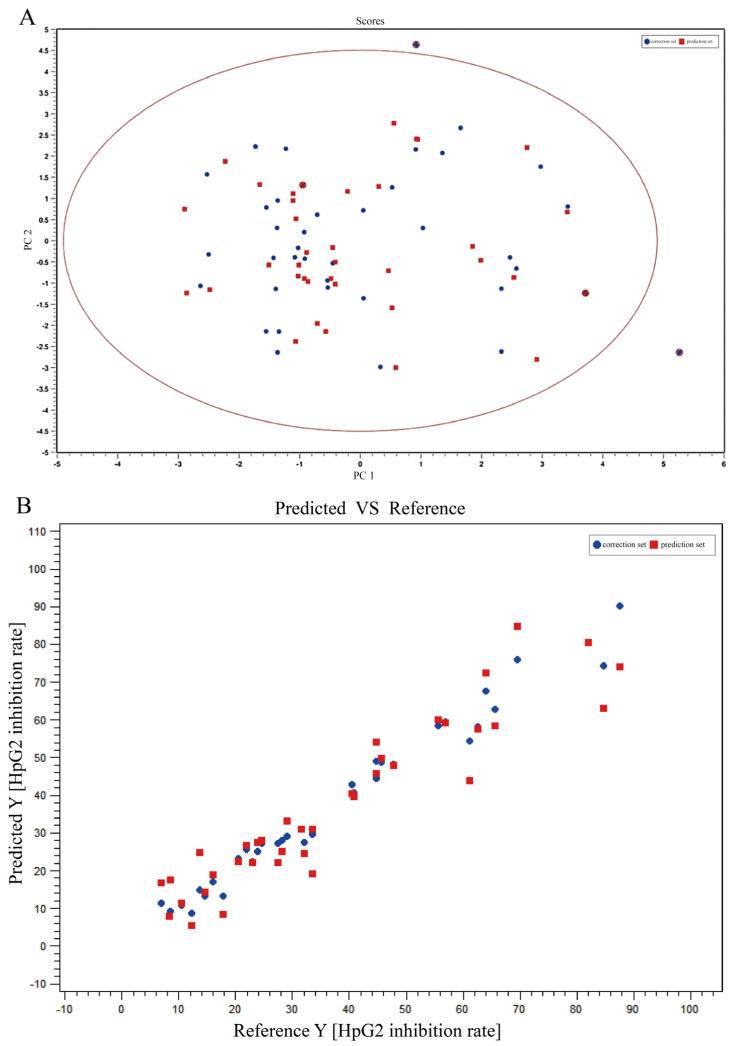
The spectral-effect relationships between the chemical composition and anti-cancer activity of *T. hemsleyanum* on HepG2 cells. PCA scores of metabolites of *T. hemsleyanum* extracts with respect to the anti-cancer IC_50_ of HepG2 (**A**); PLS modeling of metabolites of *T. hemsleyanum* extracts with respect to the anti-cancer IC_50_ of HepG2 (**B**).

The anti-cancer IC_50_ of *T. hemsleyanum* extract on HepG2 tumor cells was set as the dependent variable by ChemDateSolution software. As the independent variable, the peak list of fingerprint profiles obtained after processing by Progenesis QI (Waters Corporation, Milford, MA, USA) software was used. As shown in Figure 2, the correlations of the principal components were identified by PLS after excluding the abnormal samples (ZJ-NB and JX-JGS) by the PCA score table, and the spectral effect relationships between the chemical composition and anti-cancer effect of *T. hemsleyanum* were obtained. R^2^Y > 0.8 and Q^2^Y > 0.6 were obtained by PLS analysis, and R^2^Y gradually tends to one. Both the sample set and the validation set have the same trend, which shows that the model is useful and can be relied on. The correlation analysis indicated that there are six potential effective components related to the anti-cancer effect of *T. hemsleyanum* on HepG2 tumor cells. The fragment information of the components were shown in Table 2. Mass spectrometry was used to identify chlorogenic acid, quinic acid, catechin, kaempferol-3-O-rutinoside, and apigenin-8-C-glucoside-arabinoside, which were five of the six possible effective parts. The Q^2^Y values of these five components, which were chlorogenic acid (Q^2^Y = 0.65), quinic acid (Q^2^Y = 0.72), catechin (Q^2^Y = 0.78), kaempferol-3-O-rutinoside (Q^2^Y = 0.85), and apigenin-8-C-glucoside-arabinoside (Q^2^Y = 0.88), were all greater than 0.6 and close to 1. This indicates that these components have an inhibitory effect on the growth of HepG2 liver cancer cells, thus proving the inhibitory effect of the *T. hemsleyanum* extract on the growth of HepG2 liver cancer cells. 

### 2.2. Screening for Biomarkers of Anticancer Activity of T. Hemsleyanum in HuH-7 Hepatocellular Carcinoma Cells

#### 2.2.1. Anti-Cancer Efficacy Analysis on HuH-7 Cells

The anti-cancer effects of *T. hemsleyanum* extracts on HuH-7 hepatocellular carcinoma cells were analyzed, as shown in Table 3. Different origins of *T. hemsleyanum* showed different anti-cancer abilities. The results showed that the extracts of FJ-SM (IC_50_ = 163.96 μg/mL) appeared to have the strongest anti-HuH-7 activity, followed by JX-JGS (IC_50_ = 216.26 μg/mL), ZJ-FY (IC_50_ = 228.37 μg/mL), ZJ-PQ (IC_50_ = 230.62 μg/mL), SC-CQ (IC_50_ = 248.57 μg/mL), and FJ-SM (IC_50_ = 163.96 μg/mL). However, the inhibitory effect on HuH-7 tumor cells was weaker for *T. hemsleyanum* extracts of AH-HS (IC_50_ = 1595.99 μg/mL), GX-SC (IC_50_ = 1216.54 μg/mL), and GS-LS (IC_50_ = 938.08 μg/mL).

#### 2.2.2. Spectrum-Effect Relationship of *T. hemsleyanum* for Anti-Cancer with HuH-7

**Figure 3:**
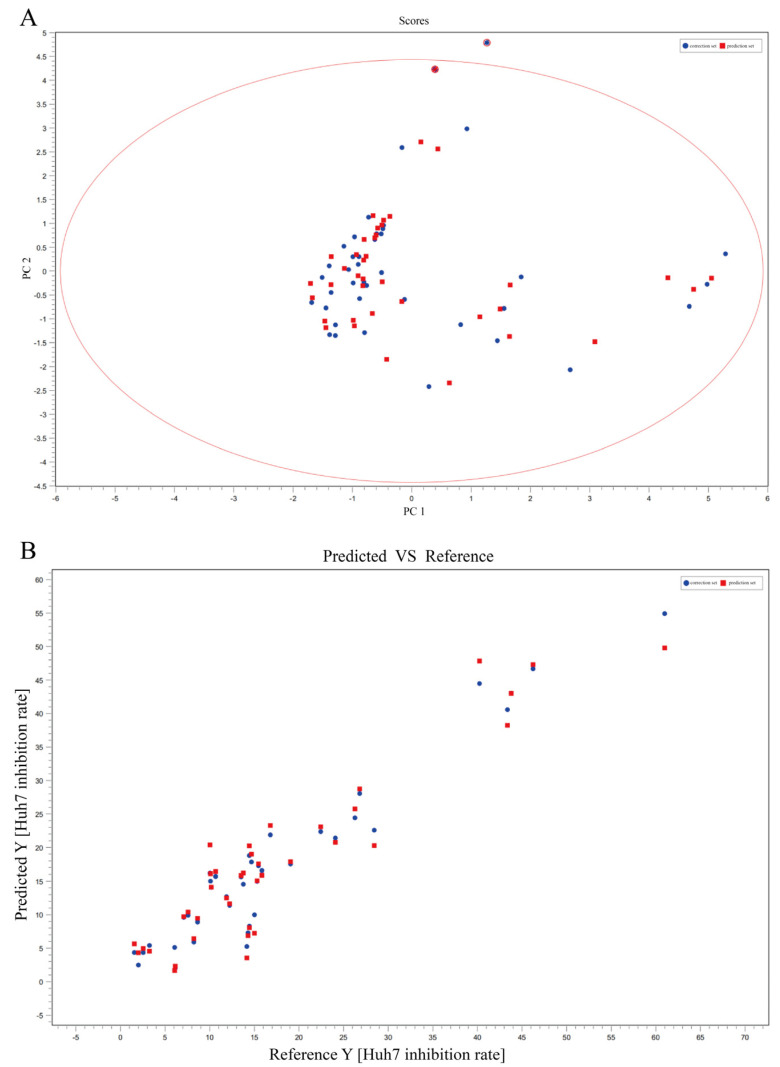
The spectral-effect relationships between the chemical composition and anti-cancer activity of *T. hemsleyanum* on HuH-7 cells. PCA scores of metabolites of *T. hemsleyanum* extracts with respect to the anti-cancer IC_50_ of HuH-7 (**A**); PLS modeling of metabolites of *T. hemsleyanum* extracts with respect to the anti-cancer IC_50_ of HuH-7 (**B**).

As shown in Figure 3, the correlations of the principal components of *T. hemsleyanum* were identified using PLS after excluding the abnormal samples (ZJ-NB, JX-JGS, and ZJ-PQL) by the PCA score table. The spectral effect relationships between the chemical composition and anti-cancer activity of *T. hemsleyanum* were determined. The PLS analysis resulted in R^2^Y > 0.8 and Q^2^Y > 0.8, with R^2^Y gradually approaching 1. The validation set showed the same trend as the sample set, indicating that the model was practical and predictable. The spectrum-effect relationship demonstrated that chlorogenic acid (Q^2^Y = 0.82), quinic acid (Q^2^Y = 0.85), linolenic acid (Q^2^Y = 0.85), and kaempferol-3-rutinoside (Q^2^Y = 0.86) were effective ingredients in *T. hemsleyanum* for promoting necrosis and apoptosis of HuH-7 cells (Table 4).

### 2.3. Screening for Biomarkers of Anti-Inflammatory Activity of T. hemsleyanum on Inflammatory Model Damage in RAW264.7 Cells

#### 2.3.1. *T. hemsleyanum* Extracts and LPS Exhibits Cytotoxicity on RAW264.7 Cells

**Figure 4:**
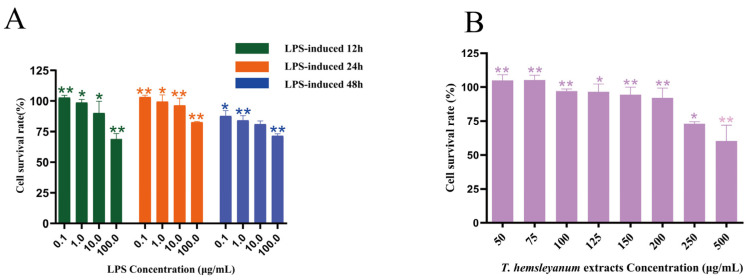
The cytotoxicity of *T. hemsleyanum* and LPS on RAW264.7 cells. Cytotoxicity of *T. hemsleyanum* extracts on RAW264.7 macrophages (**A**), * *p* < 0.05, ** *p* < 0.01; cytotoxicity of LPS on RAW264.7 macrophages (**B**), * *p* < 0.05, ** *p* < 0.01.

The CCK-8 assay was conducted to investigate the cytotoxicity of *T. hemsleyanum* and LPS on RAW264.7 cells. The methanolic extract of *T. hemsleyanum* was tested at final concentrations of 50, 100, 150, 200, 250, and 500 μg/mL on RAW264.7 cells. The results showed that the survival rate of RAW264.7 cells was above 90% after 48 h incubation with samples in the concentration range of 0–200 μg/mL. RAW264.7 cells were also treated with different concentrations of LPS (0.1, 1.0, 10.0, 100 μg/mL) for 12, 24, and 48 h. As shown in Figure 4B, the cell viability rate significantly decreased in a dose–time-dependent manner, with the viability rate after 24, 48, and 72 h exposure to LPS decreasing even at the lowest concentration (0.1 μg/mL). However, the survival rate of RAW264.7 macrophages was 90% when LPS-induced RAW264.7 cells were exposed to low concentrations in the time range of 12–24 h (*p* < 0.05). Therefore, *T. hemsleyanum* extracts with a final concentration of 200 μg/mL, a LPS concentration of 1 μg/mL, and an induction time of 12 h were chosen as the optimal conditions for the experiments to reduce the effect of LPS itself on RAW264.7 cell death and to avoid errors in subsequent experiments. This parameter selection method reflects the real cell survival rate and lays the foundation for further inflammation modeling and pharmacodynamic activity assays of RAW264.7 cells.

#### 2.3.2. Establishment of a Model of LPS-Induced Inflammation in Macrophage RAW264.7 Cells

**Figure 5:**
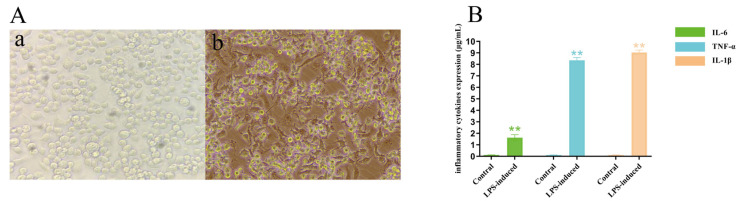
LPS-induced RAW264.7 cell inflammation model. Normally grown cells (a) and cell morphology after LPS treatment (b) (200×) (**A**); Expression of proinflammatory factors in RAW264.7 cells were induced by LPS (**B**), ** *p* < 0.01.

The image of microscopic cells indicated the morphology of normal cells and LPS-treated RAW264.7 cells (Figure 5A,B). Cellular morphology reflected typical characteristics of RAW264.7 cells after LPS processing, with a form of narrow and hypertrophic cell body appearing with prismatic structures, abundant cytoplasm, and a large nucleus (Figure 5A,B). However, normally growing cells appear uniform and have a complete oval cell morphology (Figure 5A). In addition, the expression of characteristic inflammatory cytokines in RAW264.7 cells after LPS induction was measured, and the expression of three inflammatory cytokines (IL-1β, IL-6, and TNF-α) was significantly increased (Figure 5B). Compared with normal cells, IL-1β (R = 8.35) and TNF-α (R = 9.04) expression was increased 8–9-fold, while IL-6 (R = 1.62) inflammatory factor expression was not significant. This may be related to the different sensitivity of inflammatory cytokines in the LPS-induced inflammatory signaling pathway. The changes in cell morphology and increased expression of inflammatory cytokines indicated that the inflammation model of LPS-induced RAW264.7 macrophages was feasible.

#### 2.3.3. Effect of *T. Hemsleyanum* Extract on the Secretion Function of Pro-Inflammatory Factors in an Inflammatory Model

**Figure 6:**
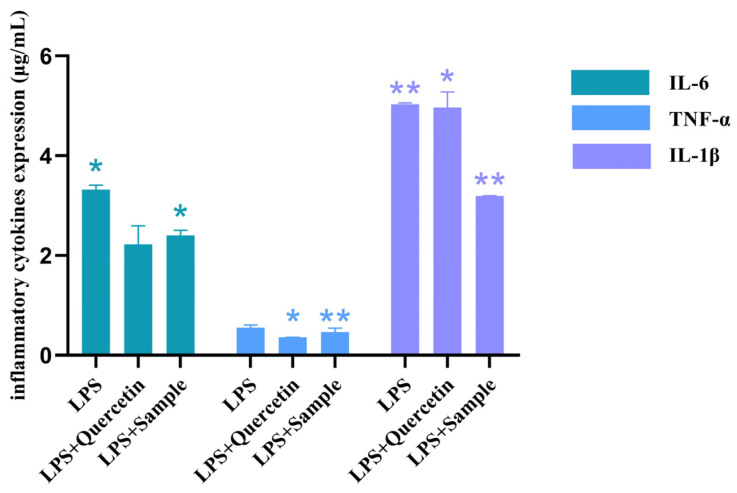
Effects of cloverleaf on the expression of inflammatory factors in a LPS-induced cell model (* *p* < 0.005, ** *p* < 0.001).

In this experiment, compound quercetin was selected as a positive control. Additionally, the expression levels of all three inflammatory cytokines were significantly decreased by quercetin (200 μg/mL) and methanolic extract samples of *T. hemsleyanum* (200 μg/mL). This indicated that *T. hemsleyanum* samples had an improved effect on the LPS-induced RAW264.7 inflammation model (Figure 6). Compared with inflammatory cytokines IL-6 and TNF-α, the expression of LPS-induced inflammatory cytokine IL-1β in RAW264.7 cells decreased by 1.10 and 0.98 after treatment with quercetin and *T. hemsleyanum*, respectively. Therefore, we chose interleukin IL-1β for the validation of subsequent experiments.

Compared with the LPS-induced inflammation model of RAW264.7 cells (IL-1β expression = 18.75), the methanolic extract of *T. hemsleyanum* from different origins showed significant improvement on the LPS-induced inflammation model of RAW264.7 cells (IL-1β, E = 3.00–16.00). As shown in Table 5, the anti-inflammatory effects of the methanolic extracts from these four origins were excellent in ZJ-HZ (R = 3.13), ZJ-FY (R = 3.39), ZJ-SX (R = 3.61) and ZJ-SX-SC (R = 3.86). This indicates that the anti-inflammatory efficacy of *T. hemsleyanum* from the Zhejiang production area is remarkable. These findings suggest that *T. hemsleyanum* exerts antiproliferative and proapoptotic effects against LPS-induced RAW264.7 macrophage cells, mediated by the downregulation of the inflammatory cytokine IL-1β.

#### 2.3.4. Spectrum-Effect Relationship Associated with the Inflammatory Factor IL-1β in the LPS-Induced Inflammation Model

**Figure 7:**
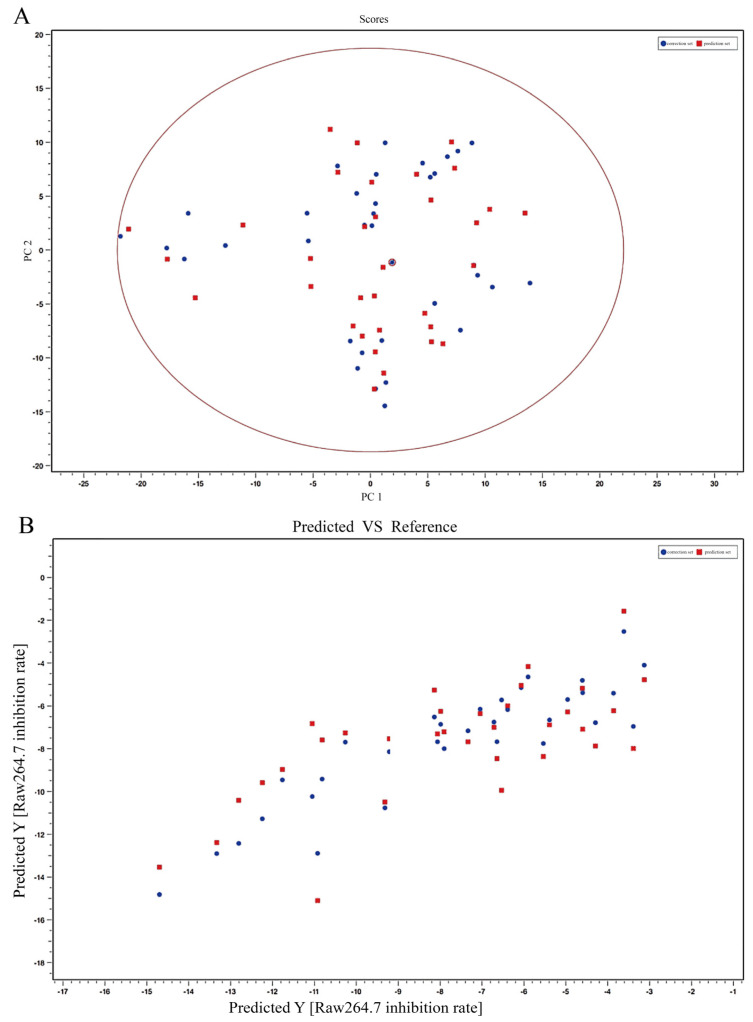
The spectral-effect relationships between the chemical composition and anti-inflammatory activity of *T. hemsleyanum* extracts from different origins on IL-1β expression in the LPS-induced RAW264.7 inflammation model. PCA scores of metabolites of *T. hemsleyanum* extracts with respect to the IL-1β expression (**A**); PLS modeling of metabolites of *T. hemsleyanum* extracts with respect to the IL-1β expression (**B**).

In this spectral efficiency model, the expression of the IL-1β factor in the LPS-induced inflammation model of RAW264.7 cells using *T. hemsleyanum* extract was set as the dependent variable. The peak list of fingerprint profiles obtained was set as the independent variable. As shown in Figure 7, the correlations of the principal components were identified by PLS after excluding the abnormal samples (ZJ-NB, JX-JGS, and ZJ-PQL) by the PCA score table, and the chemical composition relationship associated with the inflammatory factor IL-1β in the LPS-induced inflammation model was obtained. Based on PLS modeling analysis, seven relevant principal components were obtained: chlorogenic acid (Q^2^Y = 0.15), quinic acid (Q^2^Y = 0.44), kaempferol 3-rutinoside (Q^2^Y = 0.44), apigenin-8-C-glucoside (Q^2^Y = 0.27), linolenic acid (Q^2^Y = 0.16), quercetin (Q^2^Y = 0.36), and rutinum (Q^2^Y = 0.43) (Table 6). However, whether other components of *T. hemsleyanum* have anti-inflammatory efficacy on the LPS-induced inflammation model of RAW264.7 cells and its mechanism of correlation need to be further investigated.

#### 2.3.5. Docking Study

Table 7 depicts the docking scores and interacting residues of each compound against the active site of IL-1β. Generally, most of the compounds’ scores were between −4 and −6, which meant they docked well. However, ala’ score was bigger than 0 and showed a bad binding performance.

**Figure 8:**
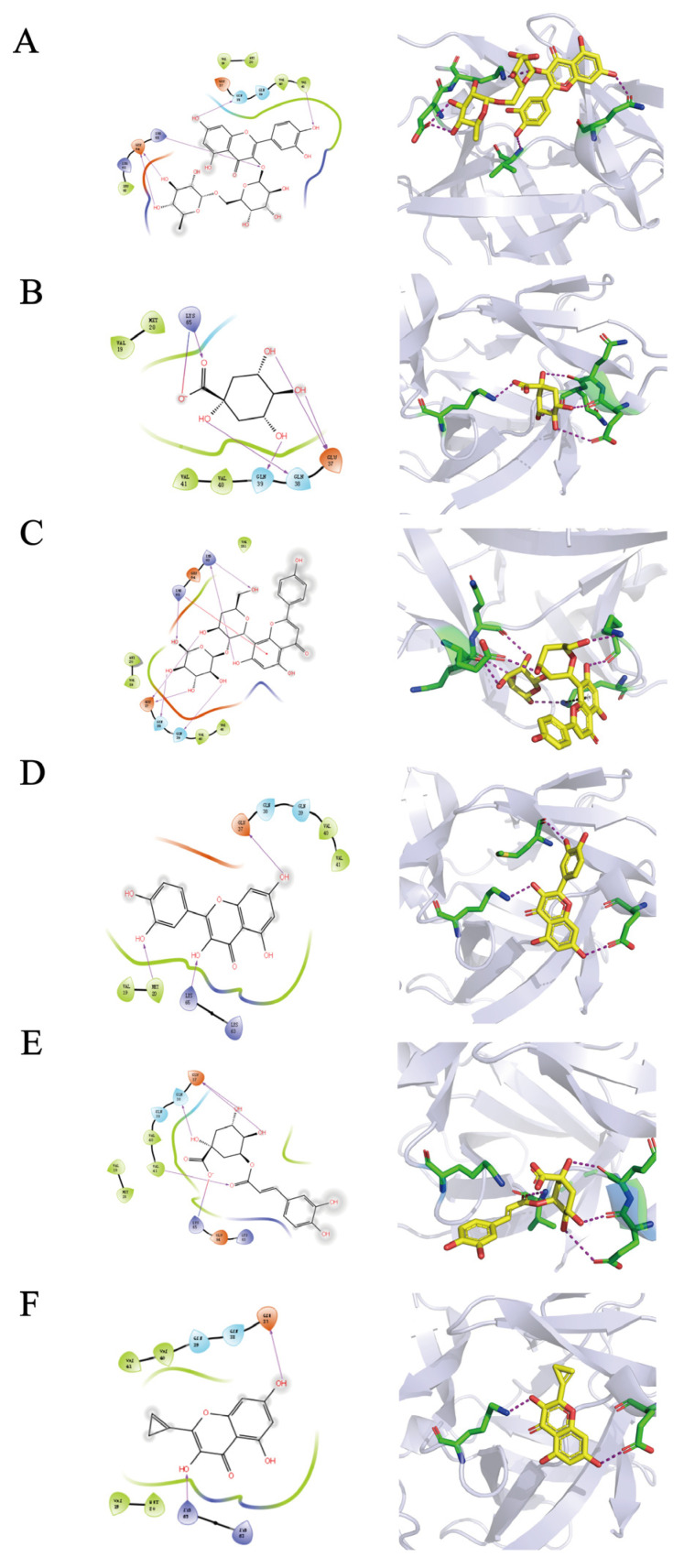
2D and 3D interaction diagrams of compounds in the active site of IL-1β. (**A**) rutin; (**B**) quinic acid; (**C**) vitexin; (**D**) quercetin; (**E**) chlorogenic acid; (**F**) Kaempferol.

Rutin showed the highest docking score against IL-1β; its interaction diagram revealed that hydrogen bonds formed between hydroxyls on aromatic rings and residues in the binding pocket constituted the main interaction force. Gln38, Val41, Glu64, and Lys65 were the key residues that were located at the opening of the pocket (Figure 8A). Due to the small space volume of quinic acid, it went deep into the pocket and showed favorable binding. Its interaction force consisted of hydrogen bonds and a salt bridge. Residues Glu37, Gln38, and Gln39 formed a stable interaction with four hydroxyls on the aromatic ring. Lys65 not only built a hydrogen bond but also a salt bridge with quinic acid’s carboxyl (Figure 8B). Figure 8C showed that the main interaction force in the complex of vitexin and pocket was hydrogen bonds as well. Glu37, Gln38, Gln39, Lys63, and Lys65 formed seven hydrogen bonds with half of the vitexin in the opening of the pocket; the other half of the vitexin stuck out of the pocket. Furthermore, Lys65 built a Pi-cation with the middle ring of vitexin. The interaction force between quercetin and the binding site consisted of hydrogen bonds completely. The interaction diagram (Figure 8D) was similar to quinic acid’s diagram to some extent and showed the formation of three hydrogen bonds with the residues Met20, Glu37, and Lys65. As shown in Figure 8E, chlorogenic acid had four hydrogen bonds with Glu37, Gln38, and Val41 and one salt bridge with Lys65. Kaempferol only built two hydrogen bonds with residues Glu37, and Lys65, which are located in the bottom of the pocket (Figure 8F). Generally, interaction diagrams showed that Glu37, Gln38, Gln39, and Lys65 were the key residues of the binding site. And the interaction force was mainly composed of hydrogen bonds and a small amount of salt bridges and pi-cations.

Based on the results of docking studies, we speculated that these compounds may inhibit the binding of the IL-1R1 receptor after binding to IL-1β at specific sites, thereby inhibiting the downstream pathway.

## 3. Discussion

### 3.1. Quality-Oriented UPLC-Q-TOF-MS Metabolite Fingerprint

Metabolite fingerprinting is a comprehensive, comparative, non-targeted approach to characterize biological processes at the metabolite level and to identify metabolites as biomarkers of whole individual developmental processes [25,26]. Traditionally, spectrum-effect relationship analysis based on ultra-performance liquid chromatography (UPLC) fingerprint data and activity studies has screened bioactive markers of traditional Chinese medicine (TCM) [27,28,29]. Mass spectrometry (MS), typically hyphenated with gas or liquid chromatography (GC-MS and LC-MS, respectively), avoids the need for complex sample pretreatment and the loss of targeted and non-targeted compounds and is one of the central tools in metabolite fingerprinting [30]. Based on this, this study used UPLC-Q-TOF-MS to identify *T. hemsleyanum* fingerprints in different habitats. However, the levels of compounds from different regions varied greatly, and the difference in their active components may be linked to the differences in their anti-cancer and anti-inflammatory effects. This makes subsequent spectral effect analysis more accurate.

### 3.2. Variation on Anti-Cancer and Anti-Inflammatory Effects of T. hemsleyanum from Different Origins

Plants have contributed extensively to the development of modern medicine for thousands of years and continue to play an important role in drug discovery [31]. Compared to other treatments, about 80 percent of the world’s population is treated with herbs or plant extracts, suggesting that plant remedies may be a better choice [32]. Modern pharmacological studies have shown that *T. hemsleyanum* has a strong anti-cancer and anti-inflammatory effect [1,2]. For example, *T. hemsleyanum* exhibits excellent anticancer ability in vitro and in vivo and initiates apoptosis by triggering key apoptotic executors downstream of the cellular pathway to make cells more responsive to death signals [33]. In addition, *T. hemsleyanum* differentially attenuated the increase of serum inflammatory factors interleukin (IL)-17 and IL-6 levels, which can significantly reduce the level of serum inflammatory [34]. Our study found that *T. hemsleyanum* significantly inhibited the proliferation rates of HepG2 and HuH-7 cells, this result is consistent with those of other studies and may contribute to the development of *T. hemsleyanum* for the pharmacological treatment of hepatoblastoma or hepatocellular carcinoma [35,36,37]. The anti-inflammatory results showed that the expression of the inflammatory factor IL-1β in the LPS-induced inflammation model treated with *T. hemsleyanum* extract was significantly lower compared with the LPS-induced cellular inflammation model [4,38]. This is while the expression and changes of the other two inflammatory factors, IL-6 and TNF-α, were not significant compared with the positive control, quercetin. This may be related to the different sensitivity of inflammatory factors in the LPS-induced inflammatory signaling pathway. However, the IC_50_ analysis revealed a wide variation in the anti-cancer and anti-inflammatory effects of *T. hemsleyanum* from different origins, which revealed the varying quality of *T. hemsleyanum.*

### 3.3. Screening Biomarkers of T. hemsleyanum by the Spectrum-Effect Relationship Method and Chemometric Analysis

Comprehensive research on the quality of traditional Chinese medicine (TCM) and establishment of the quality standards are deemed as a complicated and systematic task due to its highly complex chemical composition [39,40,41,42]. The TCM spectrum-effect relationship is an essential tool to determine the safety and efficacy of TCM in use by revealing the interrelationship between the chemical components contained in TCM and the efficacy through the interrelationship between fingerprinting and the efficacy of the medicine [43]. Through PCA, PLSR, and various statistical methods, the relationship between fingerprint characteristic peaks and drug efficacy was established to find the substance with real drug efficacy [28]. At present, the spectrum-effect relationship has been widely used in TCM research, including the study of single medicinal substances in TCM [44], famous traditional formulas [45,46], and processing mechanisms [47], providing a more scientific basis for quality control of TCM. The biomarkers of TCM refer to the intrinsic chemical substances closely associated with the functional properties that exist in the raw materials and products of TCM and can be used as indicators for quality control of TCM to embody safety and effectiveness [48]. However, the biomarkers of *T. hemsleyanum* for prevention and treatment of cancer and inflammation are not entirely clear. Therefore, this work aims to investigate the biomarkers of *T. hemsleyanum* using the spectrum-effect relationship method and chemometric analysis. The results showed that six active components of *T. hemsleyanum* extracts were associated with the inhibition of proliferation of HepG2 tumor cells, six major components were associated with the anti-cancer effect of HuH-7 tumor cells, and four of these chemical components were identified. There are seven main components related to the expression of inflammatory factor IL-1β in the RAW264.7 inflammation model of macrophages. Therefore, this study suggests that four compounds, chlorogenic acid, quinic acid, kaempferol-3-O-rutinoside, and apigenin-8-C-glucoside-arabinoside, can be selected as quality markers for *T. hemsleyanum*. This method makes up for the deficiency of separating the chemical composition from the medicinal effect. It provided a scientific and experimental basis to elucidate the chemical profiles and biomarkers of *T. hemsleyanum*.

## 4. Materials and Methods

### 4.1. Reagents and Materials

Methanol (MeOH), formic acid, acetonitrile, ethanol (all liquid chromatography–mass spectrometry (LC–MS) grade), dulbecco’s modified eagle medium/nutrient mixture F12 (DMEM/F-12), penicillin-streptomycin (100×), phosphate buffered saline (PBS), fetal bovine serum, and trypsin were obtained from Thermo Fisher Scientific (Waltham, MA, USA). Lipopolysaccharide (LPS) was purchased from Sigma-Aldrich (Shanghai, China). Cell Counting Kit-8 (CCK-8) reagent was purchased from Vazyme Biotech (Nanjing, China). The reverse transcription kit and Real-time Quantitative polymerase chain reaction (RT-PCR) kit are purchased from TOYOBO Life Sciences Company (Shanghai, China). RNA extraction kits are available at Clpbio (Montclair, CA, USA), and RNA-Quick Purification Kit was purchased from Shanghai Yishun Biotechnology company (Shanghai, China). 

*T. hemsleyanum* samples were harvested from Zhejiang, Anhui, Fujian, Guizhou, Guangxi, Guangdong, Jiangxi, and Sichuan Province, China. The sources of batches of samples are shown in Supplementary Table S1. The voucher specimens were deposited in the Key Laboratory of Plant Secondary Metabolism, Zhejiang Sci-Tech University, China (No. 2021011–42).

### 4.2. Extraction

The dried powder *T. hemsleyanum* was extracted with 80% aqueous methanol using ultrasound-assisted extraction (UAE) with an ultrasonic power setting of 200 W, 40 Hz, at 60 °C for 30 min. The combined filtrates were filtered and then rotary evaporated until there was no alcoholic smell. Finally, freeze-dried to dry cream extraction, stored in the refrigerator at 4 °C.

### 4.3. Liquid Chromatography Tandem Mass Spectrometry (LC–MS/MS) Analysis for Metabolite Identification

About 0.01 g of root extract was homogenized by adding 10 mL of MeOH/H2O (70:30, *v*/*v*), centrifuged for 5 min, and sonicated for 20 min. Then the supernatant was centrifuged at 10,000 rpm for 10 min and passed through a membrane with a pore size of 0.22 μm. The filtrate was transferred to sample bottles for LC-MS/MS analysis.

Chromatographic separation was achieved using an Accucore BEHC_18_ column (1.7 μm, 2.1 mm × 100.0 mm; Waters Corporation, Milford, MA, USA). The column temperature and flow rate were set at 30 °C and 0.3 mL·min^−1^, respectively. The column temperature was 30 °C and the flow rate was 0.3 mL·min^−1^. The injection volume of the column was 2 μL and the detection wavelength was 200~600 nm, respectively. The mobile phase consisted of 0.1% formic acid (A) and acetonitrile (B) with a gradient elution program of 95% A (0–1 min), 95–90% A (1–3 min), 90–80% A (3–6 min), 80–30% A (6–16 min) and 30–0% A (16–20 min).

An electrospray ionization source (ESI) was used to connect the above UPLC system to a Synapt G2 mass spectrometer detector (Waters Corporation, Milford, MA, USA). Argon was used as the desolvation and collision gas. The full-scan data ranged from 70 to 1200 Da; the source temperature was 120 °C; the capillary voltage was 2.6 kV; and the sample cone was 40 V. The low collision energy was set to 2 eV, and the high collision energy was steadily increased from 20 eV to 45 eV. The desolvation temperature was set at 450 °C with a desolvation gas flow of 800 L·h^−1^.

### 4.4. Assessing the Effect of T. hemsleyanum Extract on Cancer Cell Proliferation

#### 4.4.1. Cell Culture

The human hepatocellular carcinomas (HepG2 cell line) were donated by Liu Kuancheng’s research group at Sun Yat-Sen University (Guangzhou, China), and the human hepatocellular carcinomas (HuH-7 cell line) were donated by Li Gongchu’s research group at Zhejiang Sci-Tech University (Hangzhou, China). The cells were cultured in Dulbecco’s Modified Eagle Medium/Nutrient Mixture F12 (DMEM/F-12, ThermoFisher Scientific, Waltham, MA, USA) and supplemented with 10% fetal bovine serum (ThermoFisher Scientific, Waltham, MA, USA), 5 mL penicillin–streptomycin (100×, ThermoFisher Scientific, Waltham, MA, USA) under standard conditions (37 °C, 5% CO_2_).

#### 4.4.2. Cell Viability Detected by CCK-8 Assay

The 100 μL of cell suspension (1 × 10^5^/mL) was inoculated into 96-well culture plates, and 100 μL of PBS was added around the 96-well culture plates and incubated until the cells were plastered. The medium in the wells was removed, and 100 μL of the blank medium was added to the blank control group. The test groups were treated with 100 μL of samples at final concentrations of 0, 50, 100, 150, 200, 250, and 500 μg/mL for 24 h, and then 10 μL of MTT solution was added to continue the incubation for 2 h (6 replicate wells were set up for each dose group, and each group was repeated three times). The temperature of the microplate reader was set at 37 °C, and the absorbance was measured every 30 s for 5 min at 450 nm.

### 4.5. Assessment of the Effect of T. hemsleyanum Extract on Inflammatory Model Injury

#### 4.5.1. Cytotoxicity Assays

To evaluate the effect of *T. hemsleyanum* and LPS on the viability of RAW264.7 cells, cytotoxicity assays were performed using the Cell Counting Kit-8. Briefly, cells in the logarithmic growth phase were subjected to the passage procedure, and finally, 100 μL of cell suspension (with a cell density of 1 × 10^5^ /mL) was added to each well of the 96-well plate. 100 μL of PBS was added around the 96-well plate, and the blank control was a cell-free cell culture medium. The final concentrations of the master mix were 0, 0.1, 1, 10, and 100 μg/mL, and 6 replicate wells were added for each concentration, and each group was repeated three times. For the blank control, only 100 μL of cell culture medium was added. After incubation for 12 and 24 h, 10 μL of CCK-8 was added to each well and incubated for 2 h. The absorbance (OD) was measured at 450 nm on an enzyme marker. In addition, the concentration gradient of cytotoxicity of *T. hemsleyanum* extract on RAW264.7 was the same as that in Section 4.4.2.

#### 4.5.2. LPS-Induced RAW264.7 Cell Model

RAW264.7 cells in the logarithmic growth phase (1 × 10^5^/mL) were inoculated with 2.5 mL of 96-well culture plates and incubated in a CO_2_ incubator for 6 h. The culture medium was replaced with a LPS-containing medium, so that the LPS concentration was “Section 4.5.1”, the optimal concentration and the induction time was “Section 4.5.1” optimal time. Each group was set up with two replicate wells and replicated three times. For the blank control group, 2.5 mL of culture medium was added, and for the complete control group, 2.5 mL of culture medium containing 1 × 10 ^6^ cells was added. The RNA was extracted from the supernatant of the cells after 24 h. The internal reference gene was selected as GAPDH, and the contents of IL-6, TNF-α, and IL-1β were measured. According to the SYBR method, 2^^−△△CT^ values were calculated to evaluate the feasibility of the LPS-induced inflammation model in RAW264.7 cells.

#### 4.5.3. Effect of *T. hemsleyanum* Extract on the Secretion Function of Pro-Inflammatory Cytokines in an Inflammatory Model

LPS-induced RAW264.7 cell model in Section 4.5.2. After 12 h of LPS incubation, the culture was replaced with prepared *T. hemsleyanum* extract at 2.5 mL for each group, 2.5 mL of culture medium for the blank control group, and 2.5 mL of culture medium containing 1 × 10^6^ cells for the complete control group. Similarly, the internal reference gene GAPDH was selected to determine the content of IL-6, TNF-α, and IL-1β. According to the SYBR method, 2^^−△△CT^ values were calculated to evaluate the effect of *T. hemsleyanum* extract on the secretion of pro-inflammatory cytokines in an inflammatory model.

#### 4.5.4. Molecular Docking

The crystal structure of IL-1β was obtained from the RCSB Protein Data Bank (PDB). 4G6M(PDB ID) is the crystal structure of human IL-1beta in complex with the therapeutic antibody binding fragment of gevokizumab. PyMol was used to extract IL-1β from the whole structure. The crystal structure of IL-1β was selected based on the best resolution available. The protein preparation module of Schrodinger’s Maestro Molecular Modeling Suit was utilized for the preparation of the protein crystal structure. The SiteMap module of the Maestro molecular modeling package was used to find the potential pockets of IL-1β. Sobia Halim’s article helped to find the most possible pocket [49]. Glide was used for all the docking simulations and calculations. Maestro interface was used for visualization of the ligand-target interactions, including hydrogen bonding, ion-pair interactions, hydrophobic interactions, and the binding modes of the identified compounds.

### 4.6. Data Analysis

Mass spectrometry data were collected using MassLynx V4.2 software (Waters Corporation, Milford, MA, USA) for automatic peak identification, peak matching, peak alignment, peak extraction, peak integration, and peak normalization. The metabolites and their possible cleavage modes were identified using secondary mass spectrometry data from Unifi (Waters Corporation, Milford, MA, USA), online databases (SciFinder, ChemSpider, and PubMed), and data from previous studies. The median inhibitory concentration (IC_50_) of different samples was obtained by processing the efficacy data with GraphPad software. Unsupervised principal component analysis (PCA) was performed using ChemDateSolution software to obtain overall metabolic differences between sample regions and determine the magnitude of variation between samples within groups. The spectrum-effect relationships study combined with partial least squares (PLS) was performed to identify the chemical substance basis associated with the pharmacodynamic activity.

## 5. Conclusions

In this study, the UPLC-Q-TOF-MS fingerprints of extracts from *T. hemsleyanum* were established, and their medicinal effects were evaluated by three kinds of cell lines. Therefore, *T. hemsleyanum* appears to be a valuable natural source for developing anti-cancer and anti-inflammatory treatments. In addition, the relationships between the bioactive properties and UPLC-Q-TOF-MS fingerprints were analyzed by PCA and PLSR methods, and the main representative active compounds were finally discovered, such as chlorogenic acid, quinic acid, kaempferol 3-rutinoside, and apigenin 8-C-glucoside-arabinoside. The screened quality markers can provide a reference for establishing accurate, universal, and measurable scientific quality control and evaluation methods for *T. hemsleyanum*. The strategy might provide a valuable reference mode to elucidate the material basis of the complex system of TCM.

## Figures and Tables

**Table 1:** IC_50_ of growth inhibition of HepG2 tumor cells by *T. hemsleyanum* of different origins (*n* = 3, * *p* < 0.05, ** *p* < 0.01).

Sample Name	HepG2 IC_50_ (μg/mL)	Sample Name	HepG2 IC_50_ (μg/mL)
AH-HS	983.8 ± 0.98 **	ZJ-CA	301.5 ± 0.86 **
FJ-FZ	518.0 ± 1.62 **	ZJ-LQ	300.4 ± 1.14 **
FJ-SM	127.3 ± 2.66	ZJ-FY	150.7 ± 1.70
GZ-ZY	292.7 ± 1.05 **	ZJ-WY	531.4 ± 0.81 **
GZ-BJ	659.3 ± 1.64 **	ZJ-QDH	178.1 ± 1.17 **
GX-BS	207.3 ± 1.26 **	ZJ-LX	560.3 ± 0.86 **
GX-LS	619.4 ± 1.93 **	ZJ-HZ	335.1 ± 1.51 **
GD-SC	171.9 ± 1.15 **	ZJ-JS	249.1 ± 2.61
JX-JGS	187.5 ± 2.76 **	ZJ-SX-SC	723.8 ± 0.60 **
JX-SR	283.4 ± 2.43 **	ZJ-WZ	301.8 ± 0.87 *
SC-CQ	129.4 ± 2.37 *	ZJ-NB	466.9 ± 0.83 **
SC-SC	495.3 ± 1.04 **	ZJ-PQ-XG	377.8 ± 1.08 **
ZJ-QY	404.2 ± 0.79 **	ZJ-PQ-Y	315.1 ± 0.87 *
ZJ-SX	897.6 ± 0.76 **	ZJ-PQ-KG	98.7 ± 0.48 **

**Table 2:** Identification of chemical components of *T. hemsleyanum* metabolites associated with HepG2 hepatocellular carcinoma cells.

No.	Compound Name	Formula	MW/m/z	Rt/min	R^2^Y	Q^2^Y	Fragment Information
1	chlorogenic acid	C_16_H_18 9_	354.0951	0.80	0.88	0.65	95.9615,165.0341, 191.0492, 195.0430, 262.0531, 341.1071, 353.0871
2	quinic acid	C_7_H_12_O_6_	192.0634	0.83	0.93	0.72	95.9615, 191.0130
3	catechin	C_15_H_14 6_	290.0790	5.09	0.96	0.78	146.9597, 166.9872, 174.9493, 178.8363, 222.7850, 245.0751, 289.0650
4	kaempferol 3-rutinoside	C_27_H_30 15_	594.1585	6.64	0.97	0.85	178.8363, 248.9543, 316.9427, 403.1532, 425.1367, 429.1726, 475.1776, 593.1465
5	Apigenin-8-C-glucoside-arabinoside	C_26_H_28_O_14_	564.3201	16.51	0.97	0.88	178.8363, 249.1473, 293.2094, 432.2275, 504.3028, 563.3320
6	unknown	unknow	316.1478	20.78	0.98	0.88	166.9872, 178.8363, 249.1473, 297.1497, 316.9427, 317.1371

Note: No: numero; MW: molecular weight; Rt: retention time; R^2^Y refers to the determination coefficient of the model; Q^2^Y refers to the determination coefficient of cross-validation.

**Table 3:** IC_50_ of growth inhibition of HuH-7 tumor cells by *T. hemsleyanum* of different origins (*n* = 3, * *p* < 0.05, ** *p* < 0.01).

Sample Name	HepG2 IC_50_ (μg/mL)	Sample Name	HepG2 IC_50_ (μg/mL)
AH-HS	1595.99 ± 2.38 **	ZJ-SX	486.42 ± 1.17 **
FJ-FZ	693.00 ± 0.66 **	ZJ-PQ-KG	490.92 ± 0.98 **
FJ-SM	163.96 ± 7.77 **	ZJ-CA	646.41 ± 3.78 **
FJ-SC	851.61 ± 0.16 **	ZJ-LQ	380.66 ± 0.06 **
GZ-ZY	681.19 ± 3.77 **	ZJ-FY	650.16 ± 0.16 **
GZ-BJ	841.75 ± 0.22 **	ZJ-WL	610.84 ± 2.45 **
GZ-SC	630.91 ± 1.05 **	ZJ-WY	921.56 ± 0.65 **
GX-HC	692.04 ± 0.05 **	ZJ-QDH	726.21 ± 0.96 *
GX-BS	595.59 ± 3.20 **	ZJ-LX	817.72 ± 7.09 **
GX-SC	1216.54 ± 4.45 **	ZJ-HZ	631.32 ± 0.63 **
GX-LS	938.08 ± 9.26 **	ZJ-JS	372.99 ± 0.11 **
GD-SC	445.63 ± 4.04 **	ZJ-SX-SC	757.40 ± 0.02 **
JX-JGS	216.26 ± 1.29	ZJ-WZ	997.00 ± 2.14 **
JX-SR	706.21 ± 37.26 **	ZJ-NB	351.74 ± 0.46 **
SC-CQ	248.57 ± 0.10 *	ZJ-PQ-XG	230.62 ± 0.08 **
SC-SC	653.59 ± 0.40 **	ZJ-PQ-Y	415.28 ± 1.44 **
ZJ-QY	524.93 ± 2.94 **		

**Table 4:** Identification of chemical components of *T. hemsleyanum* metabolites associated with HuH-7 hepatocellular carcinoma cells.

No.	Compound Name	Formula	MW/m/z	Rt/min	R^2^Y	Q^2^Y	Fragment Information
1	chlorogenic acid	C_16_H_18_O_9_	354.0951	0.81	0.89	0.82	191.0492, 195.0430, 353.0871
2	quinic acid	C_7_H_12_O_6_	192.0634	1.20	0.92	0.85	87.0020, 111.0035, 128.0281, 191.0130
3	kaempferol 3-rutinoside	C_27_H_30_O_15_	594.1585	8.41	0.92	0.86	112.9822, 146.9597,174.9493, 178.8363, 222.7850, 248.9543, 316.9427, 433.1093, 593.1465
4	linolenic acid	C_18_H_30_O_2_	278.2246	19.27	0.93	0.85	128.2259, 166.9872, 178.8363, 197.9579, 249.1473, 265.1455, 271.2263, 277.2089
5	unknown		340.1877	20.15	0.93	0.85	146.9597, 166.9872, 178.8363, 249.1473, 265.1455, 334.9948, 339.1977
6	unknown		310.1698	20.78	0.93	0.85	166.9872, 178.8363, 249.1473, 265.1455, 309.1671

Note: No.: numero; MW: molecular weight; Rt: retention time; R^2^Y refers to the determination coefficient of the model; Q^2^Y refers to the determination coefficient of cross-validation.

**Table 5:** Effect of *T. hemsleyanum* on IL-1β expression in the LPS-induced RAW264.7 inflammation model (*n* = 3, * *p* < 0.05, ** *p* < 0.01).

Sample Name	Inflammatory Cytokine IL-1β Expression (μg/mL)	Sample Name	Inflammatory Cytokine IL-1β Expression (μg/mL)
AH-HS	6.53 ± 0.37 **	ZJ-FY-1	6.64 ± 0.25 **
CQ-JFS	5.39 ± 0.16 **	ZJ-FY-2	10.26 ± 0.33 **
FJ-FZ	6.39 ± 0.40 **	ZJ-FY-3	4.29 ± 0.04 *
FJ-SC	12.81 ± 0.56 **	ZJ-FY-4	11.77 ± 0.13 **
FJ-SM	5.84 ± 0.93 **	ZJ-FY-5	3.39 ± 0.16 **
GD-SC	9.32 ± 0.28 *	ZJ-HZ	3.13 ± 0.11 *
GX-BS	4.61 ± 0.79 **	ZJ-LQ	7.90 ± 0.07 **
GX-HC	14.70 ± 0.76 *	ZJ-LX	6.05 ± 0.04 **
GX-LS	4.60 ± 0.12 **	ZJ-NB	4.71 ± 0.14 **
GX-SC	9.21 ± 0.09	ZJ-PQ-KG	8.19 ± 0.12 **
GZ-BJ	16.16 ± 0.79 **	ZJ-PQ-XG	4.96 ± 0.05 **
GZ-SC	7.04 ± 0.14 **	ZJ-PQ-Y	4.71 ± 0.17 *
GZ-ZY	13.33 ± 0.37 **	ZJ-QDH	10.92 ± 0.11 **
JX-JGS	9.99 ± 0.06	ZJ-QY	10.82 ± 0.12 **
JX-SC	6.72 ± 0.15 **	ZJ-SX	3.61 ± 0.36 **
JX-SR	5.54 ± 0.06 **	ZJ-SX-SC	3.86 ± 0.06 **
SC-CQ	5.90 ± 0.09 **	ZJ-WL	10.42 ± 0.37 **
ZJ-CA	13.25 ± 0.55 *	ZJ-WY	7.33 ± 0.39 **
ZJ-DQH	7.99 ± 0.22 **	ZJ-WZ	8.14 ± 0.28 **
ZJ-FY	11.05 ± 0.96 **		

**Table 6:** Identification of chemical components of *T. hemsleyanum* metabolites associated with IL-1β expression in the LPS-induced RAW264.7 inflammation model.

No.	Compound Name	Formula	MW/m/z	Rt/min	R^2^Y	Q^2^Y	Fragment Information
1	chlorogenic acid	C_16_H_18_O_9_	354.0951	0.80	0.64	0.15	129.0136, 165.0341, 195.0430, 262.0531, 327.0864, 341.1071, 357.0960, 377.0816, 379.0777
2	quercetin	C_15_H_10_O_7_	302.0427	1.44	0.75	0.36	96.9529, 117.0156, 138.9945, 166.9872, 178.8363, 206.9831, 272.9558, 280.9307, 300.9876
3	quinic acid	C_7_H_12_O_6_	192.0634	2.14	0.81	0.44	87.0020, 111.0035, 128.0281, 191.0130
4	kaempferol 3-rutinoside	C_27_H_30_O_15_	594.1585	7.01	0.82	0.44	112.9822, 178.8363, 222.7850, 248.9543, 316.9427, 403.1532, 425.1367, 429.1726, 475.1776, 593.1465
5	rutinum	C_27_H_30_O_16_	610.1534	7.35	0.82	0.43	174.9493, 248.9543, 265.9428, 300.0258, 301.0255, 310.9286, 316.9427, 384.9264, 609.1483
6	apigenin8-C-glucoside-arabinoside	C_26_H_28_O_14_	564.3201	15.63	0.83	0.37	130.9611, 178.8363, 249.1473, 275.1978, 294.2126, 361.1941,384.9350, 504.3028, 563.3320
7	linolenic acid	C_18_H_30_O_2_	278.2246	20.88	0.83	0.16	129.9681, 146.9597, 166.9872, 178.8363, 248.8543, 249.1473, 250.1482, 277.2089

Note: No.: numero; MW: molecular weight; Rt: retention time; R^2^Y refers to the determination coefficient of the model; Q^2^Y refers to the determination coefficient of cross-validation.

**Table 7:** The docking site and interaction residues of compounds with IL-1β active sites.

Compound Name	Docking Score	Interacting Residues
Rutin	−5.918359555	Gln38, Val41, Glu64, Lys65
Quinic acid	−5.571761051	Glu37, Gln38, Gln39, Lys65
Vitexin	−5.211264998	Glu37, Gln38, Gln39, Lys63, Lys65
Quercetin	−5.203226381	Met20, Glu37, Lys65
Chlorogenic acid	−5.105666547	Glu37, Gln38, Val41, Lys65
Kaempfeorl	−4.263310555	Glu37, Lys65
Linolenic acid (Ala)	0.607761907	Arg11, Gln39

## Data Availability

The datasets used and/or analyzed during the current study are available from the corresponding author on request.

## Supplementary Materials

The following supporting information can be downloaded at: https://www.mdpi.com/article/10.3390/molecules28073021/s1. Figure S1: The representative total ion chromatography (TIC) of *T. hemsleyanum* extract; Table S1: The sources of samples.

## Sample Availability

Samples of the *T*. *hemsleyanum* are available from the authors.
