# Supplementary material for: Screening out Biomarkers of Tetrastigma hemsleyanum for Anti-Cancer and Anti-Inflammatory Based on Spectrum-Effect Relationship Coupled with UPLC-Q-TOF-MS
Source: Molecules. 2023 Mar 28;28(7):3021. doi: 10.3390/molecules28073021 (PMC10096277; doi:10.3390/molecules28073021)
Supplement: Supplementary file 1 [file molecules-28-03021-s001.zip › molecules-2263589-supplementary.pdf]

molecules

MDPI

Supplementary Material

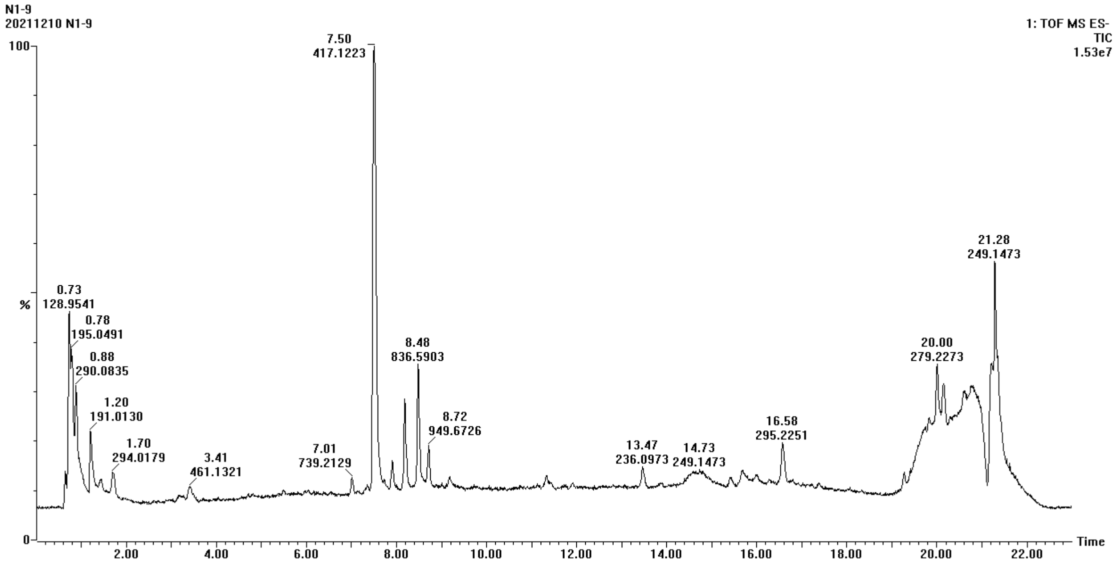

Figure S1. The representative total ion chromatography (TIC) of *T. hemsleyanum* extract.

molecules MDPI

Table S1. The sources of samples.

Sample No.	Picking or Collecting Address	Wild or Cultivated	Base Address	Period and Year of Collection
AH-HS	Huangshan, Anhui	cultivated	Sankou Town, Huangshan District, Huangshan City, Anhui Province	2021.12
FJ-FZ	Fuzhou, Fujian	cultivated	Chian Village, Dangyun Township, Yongtai County, Fuzhou City	2021.12
FJ-SM	Sanming, Fujian	cultivated	Shiliao Village, Shuixi Town, Ninghua County, Sanming City, Fujian Province	2021.12
FJ-SC	Fujian market circulation	cultivated	Fujian herbal market	2021.12
GZ-ZY	Zunyi, Guizhou	cultivated	Meitan County, Zunyi City, Guizhou Province	2021.12
GZ-BJ	Bijie, Guizhou	cultivated	Nayong County, Bijie District, Guizhou Province	2021.12
GZ-SC	Guizhou market circulation	cultivated	Guizhou herbal market	2021.12
GX-HC	Hechi, Guangxi	cultivated	Tian'e County, Hechi City, Guangxi Province	2021.12
GX-BS	Guangxi Baise market	cultivated	Guangxi herbal market	2021.12
GX-SC	Guangxi market circulation	cultivated	Guangxi herbal market	2021.12
GX-LS	Guangxi Longsheng market	cultivated	Guangxi herbal market	2021.12
GX-LY	Leye, Guangxi	cultivated	Guangxi herbal market	2021.12
GD-SC	Guangdong market circulation	cultivated	Guangdong herbal market	2021.12
JX-JGS	Jinggangshan, Jiangxi	cultivated	Jiangxi herbal market	2021.12
JX-SR	Shangrao, Jiangxi	cultivated	Yufeng Village, Huaiyu Township, Yushan County, Shangrao City, Jiangxi	2021.12
SC-CQ	Chongqing, Sichuan	cultivated	Wanzhou District, Chongqing	2021.12
SC-SC	Sichuan market circulation	cultivated	Sichaun herbal market	2021.12
ZJ-QY	Qingyuan, Zhejiang	cultivated	Qingyuan County, Lishui City, Zhejiang Province	2021.12
ZJ-SX	Shaoxing, Zhejiang	cultivated	Yujiayan Village, Keqiao District, Shaoxing City, Zhejiang Province	2021.12

ZJ-PQ	Puqian, Zhejiang	cultivated	Puqian Village, Yuhang District, Hangzhou City, Zhejiang Province	2021.12
ZJ-CA	Chunan, Zhejiang	cultivated	Chun'an County, Hangzhou City, Zhejiang Province	2021.12
ZJ-LQ	Longquan, Zhejiang	cultivated	Badu Town, Longquan City, Lishui City, Zhejiang Province	2021.12
ZJ-FY	Fuyang, Zhejiang	cultivated	Shiwu Village, Fuyang District, Hangzhou, Zhejiang Province	2021.12
ZJ-WL	Wenling, Zhejiang	cultivated	Wenling County, Taizhou City, Zhejiang Province	2021.12
ZJ-WY	Wuyi, Zhejiang	cultivated	Wuyi County, Jinhua City, Zhejiang Province	2021.12
ZJ-QDH	Qiandaohu, Zhejiang	cultivated	Qiandao Lake, Hangzhou, Zhejiang Province	2021.12
ZJ-LX	Lanxi, Zhejiang	cultivated	Chisong Village, Lanxi City, Jinhua City, Zhejiang Province	2021.12
ZJ-HZ	Hangzhou, Zhejiang	cultivated	Zhejaing herbal market	2021.12
ZJ-JS	Jingshan, Zhejiang	cultivated	Zhejaing herbal market	2021.12
ZJ-SX-SC	Shaoxing market circulation	cultivated	Zhejaing herbal market	2021.12
ZJ-WZ	Wenzhou, Zhejiang	cultivated	Zhejaing herbal market	2021.12
ZJ-NB	Ningbo, Zhejiang	cultivated	Huanhu East Road, Yinzhou District, Ningbo City, Zhejiang Province	2021.12
